# Radiomics in Cross-Sectional Adrenal Imaging: A Systematic Review and Quality Assessment Study

**DOI:** 10.3390/diagnostics12030578

**Published:** 2022-02-24

**Authors:** Arnaldo Stanzione, Roberta Galatola, Renato Cuocolo, Valeria Romeo, Francesco Verde, Pier Paolo Mainenti, Arturo Brunetti, Simone Maurea

**Affiliations:** 1 Department of Advanced Biomedical Sciences, University of Naples “Federico II”, 80131 Naples, Italy; arnaldo.stanzione@unina.it (A.S.); galatolaroberta@gmail.com (R.G.); valeria.romeo@unina.it (V.R.); francesco.verde2@unina.it (F.V.); brunetti@unina.it (A.B.); maurea@unina.it (S.M.); 2 Department of Clinical Medicine and Surgery, University of Naples “Federico II”, 80131 Naples, Italy; 3 Interdepartmental Research Center on Management and Innovation in Healthcare-CIRMIS, University of Naples “Federico II”, 80100 Naples, Italy; 4 Laboratory of Augmented Reality for Health Monitoring (ARHeMLab), Department of Electrical Engineering and Information Technology, University of Naples “Federico II”, 80100 Naples, Italy; 5 Institute of Biostructures and Bioimaging of the National Research Council, 80131 Naples, Italy; pierpamainenti@hotmail.com

**Keywords:** radiomics, adrenal imaging, methodological quality, evidence-based medicine

## Abstract

In this study, we aimed to systematically review the current literature on radiomics applied to cross-sectional adrenal imaging and assess its methodological quality. Scopus, PubMed and Web of Science were searched to identify original research articles investigating radiomics applications on cross-sectional adrenal imaging (search end date February 2021). For qualitative synthesis, details regarding study design, aim, sample size and imaging modality were recorded as well as those regarding the radiomics pipeline (e.g., segmentation and feature extraction strategy). The methodological quality of each study was evaluated using the radiomics quality score (RQS). After duplicate removal and selection criteria application, 25 full-text articles were included and evaluated. All were retrospective studies, mostly based on CT images (17/25, 68%), with manual (19/25, 76%) and two-dimensional segmentation (13/25, 52%) being preferred. Machine learning was paired to radiomics in about half of the studies (12/25, 48%). The median total and percentage RQS scores were 2 (interquartile range, IQR = −5–8) and 6% (IQR = 0–22%), respectively. The highest and lowest scores registered were 12/36 (33%) and −5/36 (0%). The most critical issues were the absence of proper feature selection, the lack of appropriate model validation and poor data openness. The methodological quality of radiomics studies on adrenal cross-sectional imaging is heterogeneous and lower than desirable. Efforts toward building higher quality evidence are essential to facilitate the future translation into clinical practice.

## 1. Introduction

Adrenal glands can be affected by a great variety of different disorders, including, but not limited to, acute illnesses, functional abnormalities, infectious diseases, and oncological processes, with diagnostic imaging playing a relevant role in most cases [1,2,3]. Radiologists are currently required to guide the management of incidental adrenal masses, whose characterization and differential diagnosis is complex and often challenging [4,5]. Indeed, while most adrenal lesions are benign (i.e., adrenal adenomas), there are several less-common entities that cannot be neglected (e.g., pheochromocytomas, metastases, adrenocortical carcinoma, lymphoma) [6,7]. Furthermore, adrenal adenomas can occasionally present atypical features due to the paucity of fat content, making it difficult to qualitatively differentiate them from malignancies [8,9]. In the effort to overcome the limitations of conventional image assessment and further increase the value of diagnostic imaging, a complex multi-step post-processing approach defined as radiomics has been proposed [10]. The premise behind this rapidly growing field is that medical images can be mined to extract quantitative data possibly related to the pathophysiology of a biological tissue [11]. These quantitative parameters, known as radiomics features, can then be used either alone or in combination with clinical information to build machine learning-based decision support tools and predictive models to aid physicians in the management of patients [12,13]. However, radiomics requires a robust workflow based on standardized, rigorous methods which are required to ensure the reliability and generalizability of results [14,15,16,17]. Using the radiomics quality score (RQS), a scoring system proposed by Lambin and colleagues and designed to assess the crucial steps in radiomics pipelines, previous investigations have found that radiomics studies are heterogeneous in terms of quality [18]. The aim of this study was to systematically review radiomics studies focused on adrenal disorders applications and to assess their quality using the RQS.

## 2. Materials and Methods

The present review was conducted in accordance with the Preferred Reporting Items for Systematic Reviews and Meta-analyses (PRISMA) guidelines [19] and the review protocol was registered on the International Prospective Register of Systematic Reviews (PROSPERO, registration number = CRD42021248536).

### 2.1. Search Strategy and Eligibility Criteria

A systematic search of published articles investigating radiomics applications to adrenal cross-sectional imaging was performed by two investigators on three major digital databases (PubMed, Scopus and Web of Science), with the end date set to 25 February 2021. The search strategy was based on the following terms and functions: “textural” OR “radiomics” OR “texture” OR “histogram” AND “adrenal” AND “computed tomography” OR “CT” OR “magnetic resonance” OR “MRI” OR “MR”. Duplicates were removed prior to screening titles and abstracts to identify original research dealing with the topic of interest, published in English and involving human subjects.

### 2.2. Data Collection and Study Evaluation

For a qualitative synthesis, the main characteristics of included studies were extracted from the full texts. In particular, first authorship, country and year of publication were registered. Then, details regarding study design (i.e., retrospective or prospective), general aim (i.e., differential diagnosis of adrenal masses, detection of adrenal diseases, evaluation of prognosis or characterization of adrenal disorders), mean goal (i.e., the specific purpose of the study), sample size (i.e., number of included patients and adrenal lesions) and imaging modality assessed were recorded. Thirdly, imaging analysis methods such as segmentation strategy, feature extraction software, extracted feature category and information about machine learning applications were noted. Finally, it was registered whether the study reported positive or negative results.

To assess the methodological quality of each radiomics study, two readers with previous experience in radiomics and the RQS (AS and RC) worked in consensus to evaluate the papers and thus calculate the score, with a third operator (VR) intervening to solve disagreements. The RQS consists of 16 items that explore crucial steps of a radiomics pipeline, including but not limited to imaging protocol, features extraction and handling, clinical relevance, and data openness [18]. Each of these has a different weight and can contribute positively or negatively in terms of points attributed, with −8 being the minimum and 36 being the maximum score that can be reached. The absolute score is then converted to a percentage value (with 36 = 100%). More details on each of the RQS items can be found in Table S1.

### 2.3. Statistical Analysis

Categorical data are presented as counts and percentages. The Shapiro-Wilk test was performed to verify whether the distribution of continuous variables was normal, and these variables are presented as either mean and standard deviation or median and interquartile range (IQR) according to the test results, as appropriate. The mode among evaluated studies was calculated for each RQS item. All analyses were conducted using the “stats” (v3.6.2) R package (v4.0.5).

## 3. Results

### 3.1. Literature Search

**Figure 1:**
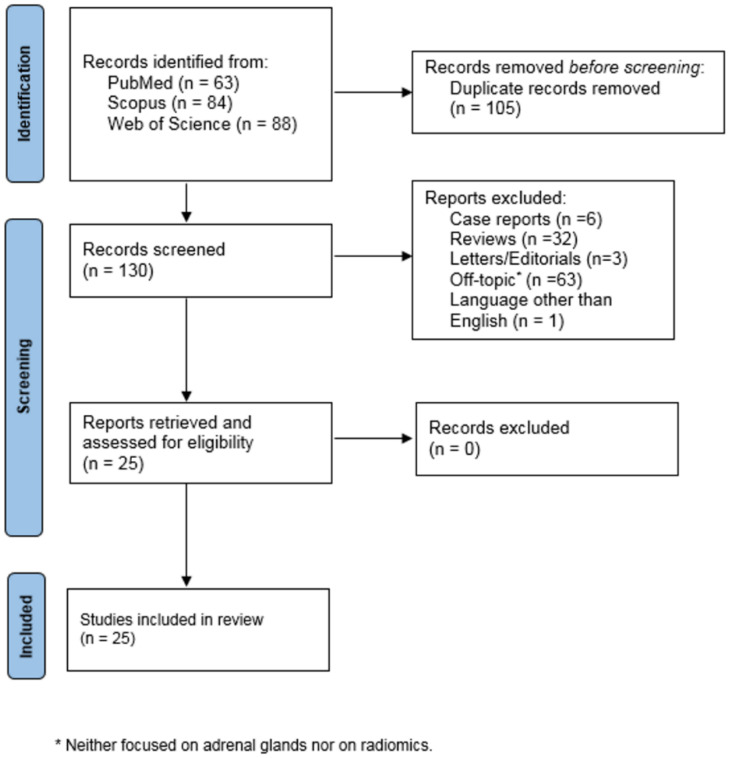
Literature search and study selection flow-chart.

The PRISMA flowchart for this study can be found in Figure 1.

Briefly, 235 records were identified, of which 105 were duplicates. After applying the selection criteria during the title and abstract screening, an additional 105 were excluded; therefore, 25 eligible articles were finally included in the present systematic review.

### 3.2. Qualitative Synthesis of Included Studies

The main characteristics of the included articles are presented in Table 1.

**Figure 2:**
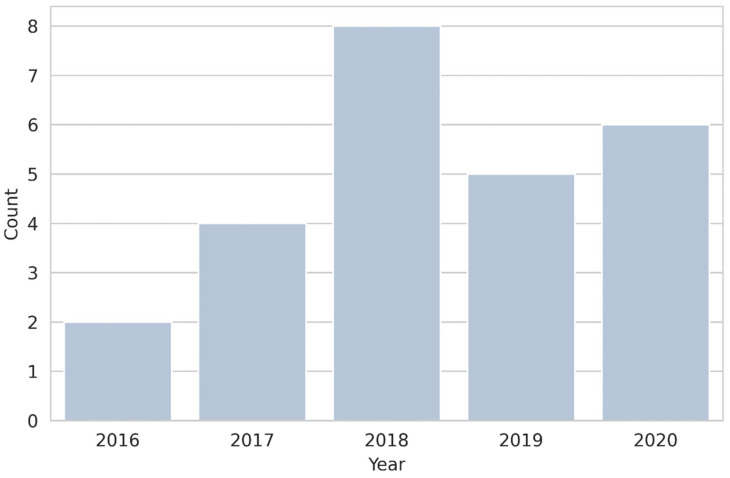
Count plot showing studies published per year.

The study design was retrospective in all cases. Out of 25, eight studies (32%) included less than 50 patients. Regarding study aim, most were focused on the differential diagnosis of adrenal masses (18/25, 72%) while only 3/25 (12%) [24,39,40] and 2/25 (8%) [21,22], respectively, investigated the role of radiomics in the evaluation of prognosis and characterization of adrenal disorders. A single article (4%) was aimed at the detection of adrenal disease (primary aldosteronism) [20] while one (4%) had multiple aims [36]. The number of included studies per publication year is illustrated in Figure 2.

CT was the most represented imaging modality (16/25, 64%), with more than half CT radiomics studies using contrast-enhanced images (11/16, 69%). A total of 4/25 (16%) MRI radiomics studies were identified, while a single article (4%) evaluated both MRI and CT images [26]. Finally, 4/25 (16%) were 2-[^18^F]FDG PET/CT studies. As for the segmentation strategy, regions of interest were predominantly annotated manually on medical images (19/25, 76%), being two-dimensional in most cases (13/25, 52%). Machine learning approaches to handle radiomics features were embraced in 12 out of 25 studies (48%). Table S2 illustrates the main characteristics of the machine learning algorithms listed in Table 1 is provided. Only one study (4%) reported negative results [40].

### 3.3. RQS Assessment

The results of the RQS assessment performed in this systematic review are reported in Table 2.

**Figure 3:**
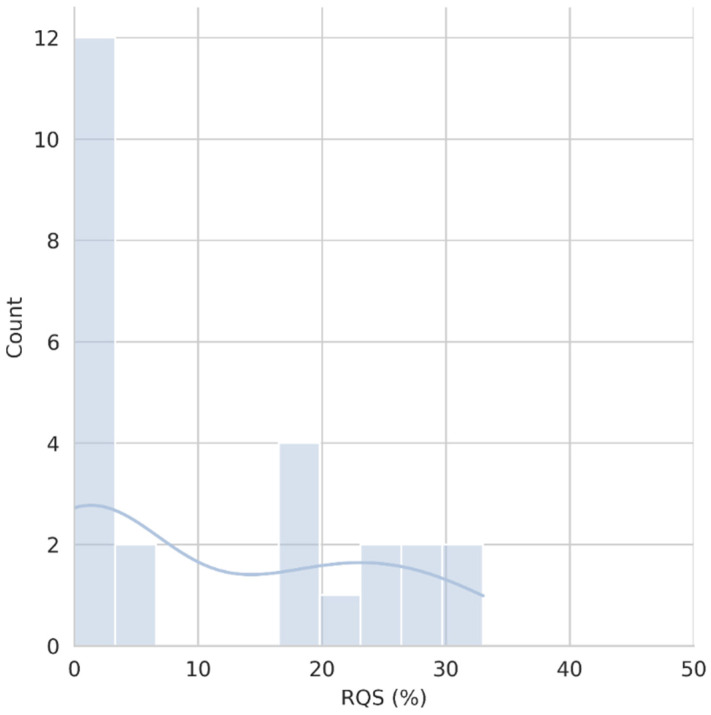
Distribution of RQS percentage score of the papers included in our review. This is presented both as a histogram (bars) and its corresponding density function (line).

**Figure 4:**
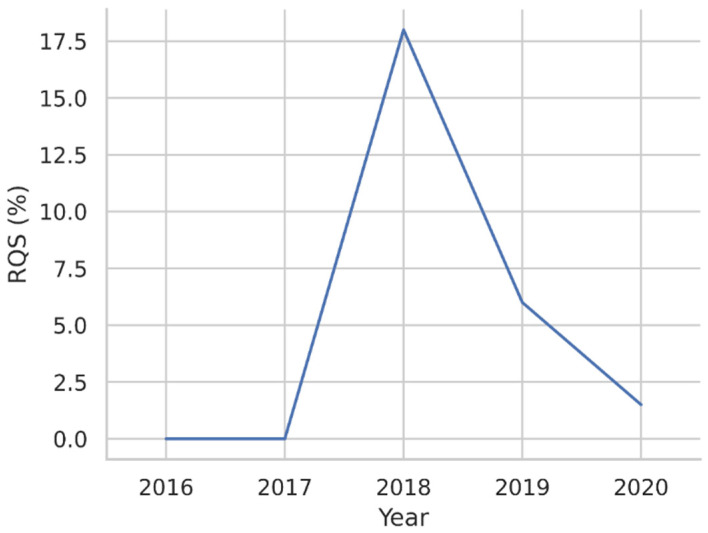
Line plot depicting the median RQS percentage score in relation to publication year.

The median total score was 2 (IQR = −5–8), corresponding to a percentage of 6% (IQR = 0–22%), with the highest and lowest scores registered corresponding respectively to 12/36 (33%) and −5/36 (0%). Figure 3 presents the distribution of RQS percentage scores in the reviewed papers while Figure 4 shows its median value by year.

Swarm plots depicting the distribution of RQS according to radiomics feature type as well as imaging modality can be found respectively in Figures S1 and S2, while the mode for each single item can be found in Table S1. Among the evaluated articles, 5/25 (20%) did not provide details regarding the imaging protocol to allow reproducibility. Most studies (18/25, 72%) did not test radiomics features robustness to segmentation variabilities. Among the included studies, radiomics features robustness testing for scanner and temporal variabilities was never performed. Feature reduction or adjustment for multiple testing was performed in 16/25 (62%) studies. Non-radiomics features were considered in 4/25 (16%) studies. Validation was missing in more than half of the included studies (13/25, 52%). Only one study performed calibration statistics (4%) [42]. The formal assessment of radiomics models’ clinical utility was never presented; similarly, there were no studies in which a cost-effectiveness analysis was performed. Finally, only one point was assigned among all studies for item 16 (open science and data) [36].

## 4. Discussion

Beyond the growing interest in radiomics and its many promises, the clinical translation of actual real-life applications must be grounded in methodologically rigorous studies and high-quality evidence. Unfortunately, previous investigations have found that the overall quality of radiomics studies is heterogenous and lower than desirable, even when exclusively focusing on works published on high-ranking scientific journals [15,45]. In this systematic review, we confirm that radiomics applications on cross-sectional adrenal imaging do not represent an exception. Indeed, the average and highest RQS scores cannot be considered satisfactory and indicate a lack in terms of methodological quality. Furthermore, our findings do not reflect a trend of quality increase in time. While “how to” guides have been published and efforts towards standardization of practice in radiomics are being carried on, most are relatively recent and therefore it might take some time before researchers begin aligning more strictly to these recommendations [46,47,48]. The evidence gathered in our systematic review suggests that three main issues might be the root cause for the overall low RQS results, namely the lack of prospectively designed studies, the absence of proper validation and the poor data openness. Indeed, a hypothetical study validating a previously published radiomics signature in a prospective setting with data (i.e., images, segmentations and code) freely available could reach a provisional score of 15/36 (42%) with just three RQS items (thus without considering additional points to be assigned). On one hand, this highlights a certain degree of strictness for the RQS system, since about half of the points are linked to three of the sixteen items. On the other hand, it should be acknowledged that studies with those characteristics could indeed have the potential to significantly contribute to advancing the field and may be considered a requirement prior to clinical translation of these models. Nevertheless, the prospective validation of previously published models can only be possible if exploratory and pilot investigations (typically retrospective in design) have been previously carried out. Thus, the actual value of the papers assessed in this systematic review may go beyond the mere score, provided that they represent a steppingstone for more robust trials. Our findings indicate that we could be at a crossroads: research group efforts should either be directed towards the validation of these preliminary studies or increase the methodological quality of works proposing new radiomics models. The issue of open data in radiomics studies has also been highlighted in other settings [49,50]. A possible solution to the problem might be represented by publicly available datasets, which could increase reproducibility and transparency but should be subject to rigorous quality controls themselves since it has been found that they might not be perfect [51,52]. However, generalizability remains one of the main endpoints and all those strategies increasing repeatability and reproducibility in radiomics are worthy of consideration [11].

The total number of included studies in this systematic review appears lower when compared to similar investigations focused on more prevalent and clinically relevant disorders, such as radiomics applications on prostate or pancreatic cancer [53,54]. However, it is in line with those reported in the evaluation of less frequent diseases (e.g., osteosarcoma) [55]. This is probably due to the fact that larger imaging datasets are more frequently available for those abnormalities which require a higher workload for radiology departments. Furthermore, the efforts of research groups are obviously driven by the perceived clinical needs to be addressed and their relevance and urgency. Nevertheless, the number of articles retrieved in each of the last three years is higher compared to the number of studies published in both 2016 and 2017, suggesting a possible trend in the interest toward adrenal cross-sectional imaging radiomics that is coherent with the rising enthusiasm on radiomics in general. However, this trend is not as strong when compared to the field of radiomics taken as a whole, which has shown a yearly growth rate of over 170% [56]. Unsurprisingly, almost the entirety of studies was focused on oncologic disorders, with a single exception [20]. Indeed, the vast majority of adrenal imaging exams are requested to investigate adrenal masses and the main field of application for radiomics is oncologic imaging [57].

Nearly half of the included studies used radiomics features to train machine learning algorithms and build classifiers for the desired task. While artificial intelligence certainly represents a valid choice to handle massive amounts of data compared with more traditional approaches (e.g., traditional statistics), it can prove complex and inscrutable when it comes to gaining clear insights on what processes are behind a certain result [58,59]. If we also consider that the sample size in the included studies was often limited, it appears understandable and agreeable that many researchers did not choose to employ artificial intelligence algorithms in their pipelines. Accordingly, the RQS does not seem to penalize or favor an approach over the other.

Segmentation remains a challenging and crucial step in radiomics pipeline, mainly because of reproducibility concerns [11]. Ideally, fast automated segmentation of the whole volume of interest would represent the optimal solution to extract representative radiomics data without time-consuming hard manual labor. However, in this systematic review, we found that two-dimensional image annotations and manual segmentation strategies were slightly preferred. Studies regarding the potential impact of segmentation strategy in cross-sectional adrenal imaging could help guide future research and reduce heterogeneity in the literature.

It is interesting to note that merely one study among those included in this review presented negative findings [40]. Publishing negative results is of paramount importance, especially in an emerging field such as radiomics, but it is often difficult and our findings seem to confirm this well-known issue [56]; scientific rigor should be the main criteria for publishing, regardless of the outcome, and it could be speculated that we might be currently missing out on high quality research for a wrong reason [60].

The present study suffers from some limitations that deserve to be discussed. Firstly, only recognized databases were searched and therefore the grey literature data may be missing from our evaluation. While this might mean that some possibly relevant studies were not assessed, it should be considered that there are no standardized methods to search the grey literature and it is often difficult to verify the reliability of its findings [61]. Therefore, we believe that our search strategy allowed us to balance comprehensiveness and accuracy, while making it easier to reproduce our results. Another limitation is that the body of the literature is small and highly heterogeneous; thus, we were not able to offer a deeper formal insight performing sub-group analyses. Nevertheless, in future endeavors to update the current systematic review, it can be hypothesized that this issue will be solved.

In conclusion, the methodological quality of radiomics studies on adrenal cross-sectional imaging is heterogeneous and lower than desirable. Efforts toward building higher quality evidence are essential to ensure that a valid and robust radiomics pipeline could in the future be translated into support decision tools to increase the value of adrenal imaging. The path is still long for research investigating adrenal radiomics applications, but it might be worth exploring.

## Figures and Tables

**Table 1:** Main characteristics of included articles.

Study ID	Year	Country	Aim	Mean Goal	Study Design	Patient Population (Number of Lesions)	Imaging Modality	Segmentation Method (Software/Algorithm)	Feature Extraction	ML	Features Type
Akai et al. [20]	2020	Japan	Detection	Localization of primary aldosteronism	Retrospective	82 (82)	Unenhanced CT	Semi-automatic, 2D (TexRAD)	TexRAD;	No	First-order
Amhed et al. [21]	2020	USA	Characterization	Prediction of Ki-67 expression in ACC	Retrospective	53 (53)	Contrast-enhanced CT	Manual, 3D (AMIRA)	PyRadiomics	No	Shape-based, first- and higher-order
Ansquer et al. [22]	2020	France	Characterization	Biological and genetic profiling of pheo	Retrospective	49 (52)	2-[^18^F]FDG PET/CT	Automatic, 3D (STAPLE)	Image Biomarker Standardization Initiative	No	Higher-order
Chen et al. [23]	2018	USA	DD	Benign vs. malignant	Retrospective	222 (222)	Unenhanced and contrast-enhanced CT	NR	NR	Yes (Bayesian classifier)	NR
Daye et al. [24]	2019	USA	Prognosis	Local progression and survival in ablated adrenal metastasis	Retrospective	21 (21)	Contrast-enhanced CT	Manual, 3D (NR)	MATLAB	Yes (support vector machine)	Higher-order
Elmohr et al. [25]	2019	USA	DD	Adenoma vs. ACC	Retrospective	54 (54)	Unenhanced and contrast-enhanced CT	Manual, 3D (AMIRA)	PyRadiomics	Yes (random forest)	Shape-based, first- and higher-order
Ho et al. [26]	2019	USA	DD	Benign vs. malignant	Retrospective	20 (23)	Unenhanced and contrast-enhanced CT and MRI 3T or 1,5T T1 IN-OUT	Manual 3D (Seg3D)	Image Biomarker Standardization Initiative	No	First- and higher-order
Koyuncu et al. [27]	2018	Turkey	DD	Benign vs. malignant	Retrospective	NR (114) *	Unenhanced and contrast-enhanced CT	Semi-automatic, 2D (AbSeg)	MATLAB	Yes (neural network)	First- and higher-order
Li et al. [28]	2017	USA	DD	Benign vs. malignant	Retrospective	223 (230)	Unenhanced CT	Manual, 2D (NR)	NR	Yes (Bayesian)	Higher-order
Li et al. [29]	2018	USA	DD	Benign vs. malignant	Retrospective	204 (210)	Unenhanced and contrast-enhanced CT	Manual, 2D (NR)	NR	Yes (Bayesian)	Higher-order
Li et al. [30]	2020	USA	DD	Benign vs. malignant	Retrospective	204 (210)	Unenhanced and contrast-enhanced CT	Manual, 2D (NR)	NR	Yes (Bayesian)	Higher-order
Liu et al. [31]	2020	China	DD	Adenoma vs. pheo	Retrospective	58 (60)	MRI 3T T1 IN-OUT, T2w	Manual, 3D (Mazda)	MaZda	Yes (support vector machine)	First-order
Nakajo et al. [32]	2017	Japan	DD	Benign vs. malignant	Retrospective	31 (35)	2-[^18^F]FDG PET/CT	Semi-automatic, 3D (Advantage Windows Workstation)	Python #	No	First-order
Romeo et al. [33]	2018	Italy	DD	Adenoma vs. non-adenoma	Retrospective	60 (60)	MRI 3T T1 IN-OUT, T2w	Manual, 3D (3D Slicer)	3D Slicer	Yes (decision tree)	First- and higher-order
Shi et al. [34]	2019	China	DD	Benign vs. malignant	Retrospective	225 (265)	Unenhanced and contrast-enhanced CT	Manual, 2D (TexRAD)	TexRAD;	Yes (support vector machine)	First-order
Schieda et al. [35]	2017	Canada	DD	Adenoma vs. RCC metastasis	Retrospective	39 (44)	MRI 3T or 1.5T T1 IN-OUT, T2w, GRE T1w pre- and post-contrast	Manual, 2D (Image J)	Image J	No	First-order
Shoemaker et al. [36]	2018	USA	DD/Characterization	Benign vs. malignant; calcified vs. non calcified; functioning vs. non functioning	Retrospective	356 (379)	Unenhanced CT	NR	NR	Yes (decision tree)	First- and higher-order
Tu et al. [37]	2018	Canada	DD	Adenoma vs. lung cancer metastasis	Retrospective	61 (76)	Contrast-enhanced CT	Manual, 2D (ImageJ)	ImageJ	No	First-order
Umanodan et al. [38]	2016	Japan	DD	Adenoma vs. pheo	Retrospective	47 (52)	MRI 3T ADC	Manual, 2D (Synapse Vincent)	Synapse Vincent	No	First-order
Wang et al. [39]	2019	China	Prognosis	Survival in primary adrenal non-Hodgkin’s lymphoma	Retrospective	19 (19) §	2-[^18^F]FDG PET/CT	Manual, 3D (LifeX package)	LifeX package	No	First- and higher-order
Werner et al. [40]	2016	Germany	Prognosis	Disease progression and survival in ACC	Retrospective	10 (10)	2-[^18^F]FDG PET/CT	Manual, 3D (Interview Fusion Workstation)	Interview Fusion Workstation	No	First- and higher-order
Yi et al. [41]	2018	China	DD	Adenoma vs. pheo	Retrospective	108 (110)	Unenhanced CT	Manual, 2D (MaZda)	MaZda	Yes (logistic regression)	First- and higher-order
Yi et al. [42]	2018	China	DD	Adenoma vs. pheo	Retrospective	265 (265)	Unenhanced and contrast-enhanced CT	Manual, 2D (MaZda)	MaZda	No	First- and higher-order
Yu et al. [43]	2020	USA	DD	Benign vs. malignant	Retrospective	125 (125)	Contrast-enhanced CT	Manual, 2D (TexRAD)	TexRAD,	No	First-order
Zhang et al. [44]	2017	China	DD	Adenoma vs. pheo	Retrospective	155 (164)	Unenhanced and contrast-enhanced CT	Manual, 2D (TexRAD)	TexRAD,	No	First-order

* reported number of images. # formulas reported in the article. § patients with adrenal and/or kidney lymphoma included. ACC: adrenocortical carcinoma, DD: differential diagnosis of adrenal masses, Pheo: pheochromocytoma, NR: not reported.

**Table 2:** Radiomics Quality Score (RQS) assessment for all included articles.

Study ID	Item 1	Item 2	Item 3	Item 4	Item 5	Item 6	Item 7	Item 8	Item 9	Item 10	Item 11	Item 12	Item 13	Item 14	Item 15	Item 16	Total	(%)
Akai et al. [20]	1	0	0	0	3	0	0	1	1	0	0	−5	0	0	0	0	1	3%
Amhed et al. [21]	1	0	0	0	−3	0	0	1	1	0	0	−5	0	0	0	0	−5	0
Ansquer et al. [22]	1	1	0	0	3	1	1	1	2	0	0	2	0	0	0	0	12	33%
Chen et al. [23]	0	0	0	0	3	0	0	0	1	0	0	2	0	0	0	0	6	17%
Daye et al. [24]	1	0	0	0	−3	0	0	0	2	0	0	2	0	0	0	0	2	6%
Elmohr et al. [25]	1	1	0	0	3	0	0	0	2	0	0	2	2	0	0	0	11	31%
Ho et al. [26]	1	0	0	0	3	0	0	0	1	0	0	−5	0	0	0	0	0	0%
Koyuncu et al. [27]	0	0	0	0	3	0	0	0	1	0	0	2	0	0	0	0	6	17%
Li et al. [28]	0	0	0	0	3	0	0	0	1	0	0	2	0	0	0	0	6	17%
Li et al. [29]	1	0	0	0	3	0	0	0	1	0	0	2	0	0	0	0	7	19%
Li et al. [30]	0	0	0	0	3	0	0	0	1	0	0	−5	0	0	0	0	−1	0
Liu et al. [31]	1	1	0	0	3	0	0	1	1	0	0	2	0	0	0	0	9	25%
Nakajo et al. [32]	1	0	0	0	−3	0	0	1	1	0	0	−5	0	0	0	0	−5	0
Romeo et al. [33]	1	0	0	0	3	0	0	0	2	0	0	2	2	0	0	0	10	28%
Shi et al. [34]	1	1	0	0	3	0	0	0	2	0	0	2	0	0	0	0	9	25%
Schieda et al. [35]	1	0	0	0	−3	1	0	0	1	0	0	−5	0	0	0	0	−5	0
Shoemaker et al. [36]	0	0	0	0	3	0	0	0	2	0	0	2	0	0	0	1	8	22%
Tu et al. [37]	1	0	0	0	−3	0	0	1	1	0	0	−5	0	0	0	0	−5	0
Umanodan et al. [38]	1	1	0	0	−3	0	0	1	1	0	0	−5	0	0	0	0	−4	0
Wang et al. [39]	1	0	0	0	3	1	0	1	1	0	0	−5	0	0	0	0	2	6%
Werner et al. [40]	1	0	0	0	−3	1	0	0	1	0	0	−5	0	0	0	0	−5	0
Yi et al. [41]	1	1	0	0	3	0	0	0	1	0	0	−5	0	0	0	0	1	3%
Yi et al. [42]	1	1	0	0	3	0	0	1	1	1	0	2	0	0	0	0	10	28%
Yu et al. [43]	1	0	0	0	−3	0	0	1	1	0	0	−5	0	0	0	0	−5	0
Zhang et al. [44]	1	0	0	0	−3	0	0	1	1	0	0	−5	0	0	0	0	−5	0

## Data Availability

Data are contained within the article or supplementary material.

## Supplementary Materials

The following supporting information can be downloaded at: https://www.mdpi.com/article/10.3390/diagnostics12030578/s1, Table S1: Radiomics quality score items and their respective scores as described by Lambin et al.; Table S2: Basic principles of the most adopted machine learning algorithms in adrenal imaging; Figure S1. Swarm plot of the RQS distribution according to imaging modality; Figure S2. Swarm plot of the RQS distribution according to feature type.
