# Supplementary material for: Radiomics in Cross-Sectional Adrenal Imaging: A Systematic Review and Quality Assessment Study
Source: Diagnostics (Basel). 2022 Feb 24;12(3):578. doi: 10.3390/diagnostics12030578 (PMC8947112; doi:10.3390/diagnostics12030578)

diagnostics MDPI

Table S1. Radiomics quality score items and their respective scores as describe by Lambin et al. *[Lambin, P., Leijenaar, R., Deist, T. et al. Radiomics: the bridge between medical imaging and personalized medicine. Nat Rev Clin Oncol 14, 749–762 (2017). https://doi.org/10.1038/nrclinonc.2017.141]*. The mode for each item in the studies included in the present systematic review is also reported.

Items	Definitions and points	Scores	Mode
**1** (Image protocol quality)	Assign one point if the imaging protocols have been properly reported (increasing reproducibility) and one further point in case public protocols have been used.	From 0 to 2	1
**2** (Multiple segmentations)	Assign one point if feature robustness to segmentation variabilities has been tested	0 or 1	0
**3** (Phantom study on all scanners)	Assign one point if feature robustness to scanner variabilities has been tested using a phantom	0 or 1	0
**4** (Imaging at multiple time points)	Assign one point if feature robustness to temporal variabilities has been tested	0 or 1	0
**5** (Feature reduction or adjustment for multiple testing)	Assign three points if either feature reduction or adjustment for multiple testing has been performed, otherwise subtract three points	-3 or +3	3
**6** (Multivariable analysis with non-radiomics features)	Assign one point if non-radiomics features have been included	0 or 1	0
**7** (Biological correlates)	Assign one point if biological correlates have been analyzed	0 or 1	0
**8** (Cut-off analyses)	Assign one point if cut-off analyses have been performed	0 or 1	0
**9** (Discrimination statistics)	Assign one point if a discrimination statistic (with statistical significance) has been performed, and one additional point in case a resampling method (e.g. bootstrapping or cross-validation) was applied too.	From 0 to 2	1
**10** (Calibration statistics)	Assign one point if a calibration statistic (with statistical significance) has been performed, and one additional point in case a resampling method (e.g. bootstrapping or cross-validation) was applied too.	From 0 to 2	0

**11**<br>(Prospective study registered in a trial database)	Assign seven points if the study design was prospective	0 or 7	0
**12**<br>(Validation)	Assign two points for internal validation, three points for a single external validation dataset, four points for either two independent external validation datasets or validation of a previously published signature or five points for three or more independent external validation datasets. Subtract five points if validation is missing.	-5 or 2 or 3 or 4 or 5	-5
**13**<br>(Comparison to "gold standard")	Assign one point if the model has been compared to the current 'gold standard'.	0 or 2	0
**14**<br>(Potential clinical utility)	Assign two points if the clinical applicability of the model has been formally assessed (e.g. decision curve analysis).	0 or 2	0
**15**<br>(Cost-effectiveness analysis)	Assign one point if cost-effectiveness analysis of the clinical application has been performed.	0 or 1	0
**16**<br>(Open science and data)	Assign one point for each of the following:<br>1) Medical images are open source<br>2) Segmentations are open source<br>3) Code is open source<br>4) Radiomics features are calculated on a set of representative segmentations (both open source)	From 0 to 4	0

Table S2. Basic principles of the most adopted machine learning algorithms in adrenal imaging.

Algorithm name	Basic functioning principle	Main advantages	Main disadvantages
**Naive Bayes**	A probabilistic model based on the Bayes' theorem with the assumption of strong (naive) independence between the features data	• Ideal for classification tasks<br>• Low computational complexity<br>• Efficient on small datasets	• Independence between features, which is unlikely in real life cases<br>• The zero-frequency problem
**Support Vector Machine**	This technique aims to identify the best hyper plans in an n-dimensional space (n= the number of features) meant as decision boundaries that help classify the data points	• Ideal for classification and regression tasks<br>• Good performance with highly dimensional data<br>• It is not affected by outliers	• Bad performance with overlapped classes<br>• It exhibits slow speed for training process
**Decision Trees**	Non-parametric methods used for constructing predictive models by learning simple decision rules inferred from the data features	• Ideal for classification tasks<br>• Easy to understand and interpret<br>• Handling of categorical data	• Tendency to overfit the training data<br>• High computational complexity
**Random Forest**	An ensemble method consisting of multiple decision trees that are trained independently to make output predictions	• Ideal for classification and regression tasks<br>• Reduced issues with overfitting<br>• Good handling on imbalanced datasets	• The features need to have strong predictive power<br>• Lack of Interpretability (black box effect)
**Logistic Regression**	A probabilistic technique used to obtain the best fitting model for the relationship between multiple predictors and a dichotomous target variable	• Ideal for classification and regression tasks<br>• Simple implementation and interpretability	• Low performance for non-linear features<br>• Sensitive to outliers

		• Reduced overfitting drawbacks	
**Artificial Neural Networks**	A subset of computing systems structured in neuronal-like multi-layered architectures with the ability to automatically process high dimensional features, capture complex patterns and perform specific operations	• Used in computer vision tasks including classification, detection, and segmentation<br>• Good generalisation and robustness of the results<br>• Independence from prior assumptions	• Lack of Interpretability (black box effect)<br>• High computational complexity

Figure S1. Swarm plot of the RQS distribution according to imaging modality.

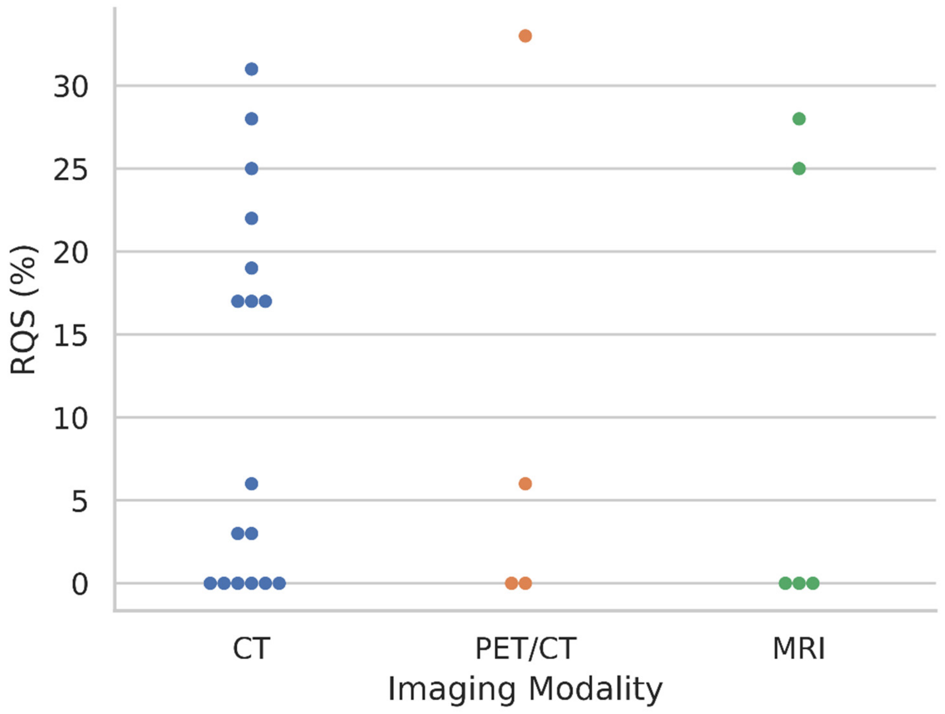

Figure S2. Swarm plot of the RQS distribution according to feature type.

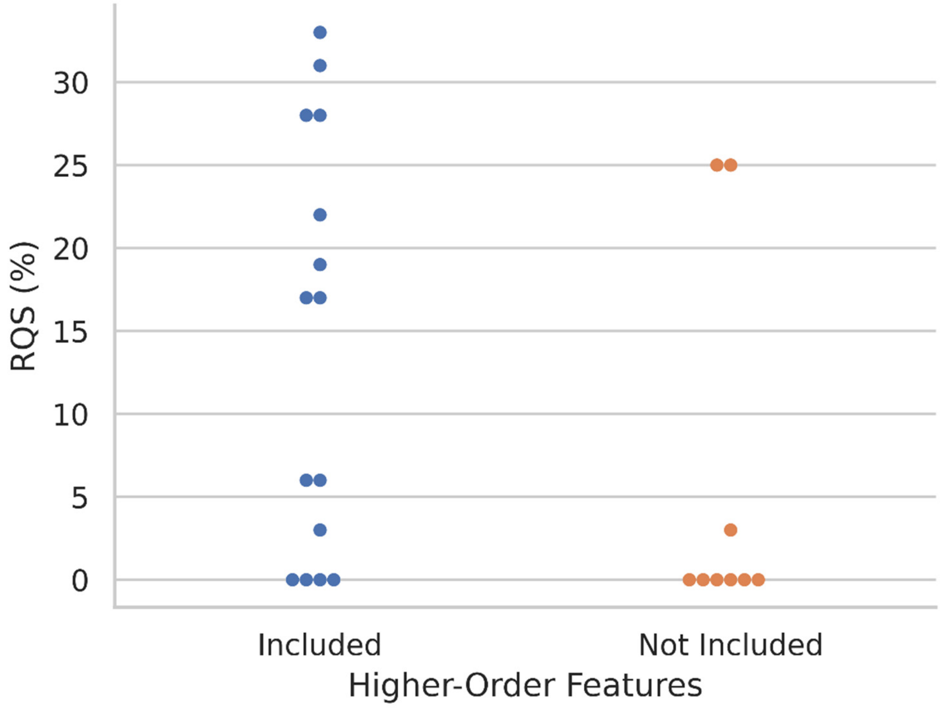

Supplement: Supplementary file 1 [file diagnostics-12-00578-s001.zip › diagnostics-1604542-supplementary.pdf]
